# Enhancing wheat genomic prediction by a hybrid kernel approach

**DOI:** 10.3389/fpls.2025.1605202

**Published:** 2025-08-01

**Authors:** Jaime Cuevas, Jose Crossa, Abelardo Montesinos-López, Johannes W. R. Martini, Guillermo Sebastiáń Gerard, Jaime Ortegón, Susanne Dreisigacker, Velu Govindan, Paulino Pérez-Rodríguez, Carolina Saint Pierre, Leonardo Abdiel Crespo Herrera, Osval A. Montesinos-López, Paolo Vitale

**Affiliations:** ^1^ División de Ciencias, Ingeniería y Tecnologías (DCIT), Universidad Autónoma del Estado de Quintana Roo, Chetumal, Quintana Roo, Mexico; ^2^ International Maize and Wheat Improvement Center (CIMMYT), Mexico-Veracruz, Edo. de México, Mexico; ^3^ Colegio de Postgraduados, Montecillos, Mexico; ^4^ Centro Universitario de Ciencias Exactas e Ingenierías (CUCEI), Universidad de Guadalajara, Guadalajara, Jalisco, Mexico; ^5^ Aardevo B.V., Nagele, Netherlands; ^6^ Facultad de Telemática, Universidad de Colima, Colima, Mexico

**Keywords:** genomics, pedigree, merging genomics and pedigree, single-environment, genotype by environment interaction

## Abstract

This study integrates genomic and pedigree data by leveraging advanced modeling techniques, aiming to enhance the predictive performance of genomic selection models by capturing complex genetic relationships through the interaction of both matrices and exploring the utility of non-linear methods, such as kernel matrices. Our goal was to improve genomic prediction accuracy by combining the pedigree-based or genetic similarity matrix (***A***) with the genomic similarity matrix (***G***). Using various wheat datasets, we performed five single-environment models and five multi-environment models that incorporated genotype-by-environment (G × E) interactions. The proposed models S5 and M5 significantly enhanced prediction accuracy by incorporating two novel symmetric kernels, ***C*** and ***P***, derived from the interaction of genomic and pedigree matrices. These hybrid kernels captured additional, independent genetic variation not explained by conventional matrices. The proposed prediction model outperformed the standard conventional models in most single-environment and multi-environment models. The genomic models with non-linear kernels were better predictors than the linear prediction models.

## 1 Introduction

Genomic Prediction (GP) (Meuwissen et al., 2001) has become a central component in breeding programs for both plant and animal species (de los Campos et al., 2013; Crossa et al., 2017; Gholami et al., 2021). In plant breeding, a breeding cycle consists of i) the creation of new variations by crossing, ii) the evaluation of the novel plant material, potentially at multiple locations and across multiple years, and iii) the selection of parents for new crosses. Genomic prediction enables the prediction of an individual’s genetic merit at early stages of the breeding program, allowing breeders to select plants for new crosses after a shorter evaluation period. The resulting reduction of the “cycle time” can lead to an increase in genetic gain per time and makes breeding programs overall more agile to react faster to new challenges (Heffner et al., 2010). This is particularly important for crops like wheat, which play a critical role in global food security.

The basic linear model for GP (Meuwissen et al., 2001) can be represented as y=μ1+g+ϵ , where the y phenotypic values are explained by a general mean or intercept μ , plus the genomic value g , and a vector of random errors ϵ . Thus, if we assume that g=Xβ , (where X is a matrix of known molecular markers and β the regression coefficients to be calculated), a group of restricted regression methods can be derived; the most commonly used of which is the Ridge Regression Best Linear Unbiased Predictor (RR-BLUP; Endelman, 2011), which assume constant variance for all coefficients of the markers, although there are other popular methods such as Bayes B and Bayes CP (Gianola et al., 2009; Habier et al., 2011; Gianola, 2013), which have other assumptions for the variance of the predictor coefficients.

Another common group derived from the basic model assumes that g is an independent random vector that behaves like a mean normal distribution, a vector of zeros, and a $\sigma_{g}^{2}K$ , where $\sigma_{g}^{2}$ is a component to be calculated and K is a relations matrix that must be known, symmetrical, and positive semi-definite relationship matrix. This matrix can be the pedigree-based relationship matrix (***A***), or the genomic relationship matrix (***G*** *)* constructed from the molecular markers that represent linear relationships between the GBLUP (Genomic Best Linear Unbiased Prediction) genotypes (VanRaden, 2008) and is equivalent to RR-BLUP. Other covariance matrices with non-linear relationships have been used in an attempt to improve genomic prediction, such as the exponential (Endelman, 2011), the t distribution (Morota and Gianola, 2014), or one of the most common ones, based on normal distribution and known as the “Gaussian kernel” (Gianola and van Kaam, 2008; de los Campos et al., 2010; Crossa et al., 2010, 2019, 2024; Montesinos-López et al., 2021).

The pedigree information (***A***) and marker data (***X***) can be integrated into a single model. One approach, proposed by Crossa et al. (2010), considers two independent random effects with their respective covariance structures: one based on the pedigree matrix and the other on a kernel matrix derived from molecular markers. Alternatively, when marker information is missing for some genotypes, but pedigree information is available, a single-step model, such as the one by Legarra et al. (2009), can be used. This model combines both sources of information into a single kernel that accounts for both pedigree and marker data.

Despite these advances, a source of information that has not been fully tapped into in the context of GP is the pedigree relationship matrix ***A*** (coefficient of parentage, CoP). The genomic relationship matrix ***G*** is considered the “realized” relationship as a contrast to the “expected” relationship, given by the pedigree relationship matrix ***A***. Therefore, when considering ***A*** simply as a less accurate description of the same information, the pedigree information may seem obsolete when genomic markers are available, from which the genomic relationship matrix ***G*** can be derived. The argument is understandable but does not consider that the additive genomic relationship is based on “identity by state”, not on “identity by descent” (Lynch and Walsh, 1998; Fernando et al., 2017). Therefore, matrix ***G*** may incorrectly model a component of the relationship, and both matrices ***A*** and ***G*** provide slightly different information. When included, pedigree matrices are typically treated as an additive component alongside genomic information, as discussed by Crossa et al. (2010).

The combined use ***A*** and ***G*** in plant breeding is crucial in leveraging both historical pedigree data and genomic marker information. This integration enhances the accuracy of genomic predictions by capturing additive genetic relationships more comprehensively, enabling more precise selection decisions and thus accelerating genetic gain.

The main objective of this study is to integrate genomic and pedigree information. Specifically, the study aims to enhance the predictive performance of genomic prediction models by (1) capturing complex genetic relationships through the combination of pedigree and genomic information, (2) exploring the utility of non-linear methods such as kernel matrices and (3) evaluating the impact of combining these sources of information in a study that attempts to improve the accuracy of genetic predictions, benefiting breeding programs by offering more precise selection tools.

We used five Single-Environment Models: 1) *Model S1* (Pedigree-Based): The phenotypic response is modeled using a multivariate normal distribution where the covariance of the random effect is defined by the relationship matrix derived from (***A***) and its variance component; 2) *Model S2* (GBLUP): Genomic effects are modeled using the GBLUP method, with the relationship matrix ***G*** derived from molecular markers that are recoded for additive effects and standardized. 3) *Model S3* (Gaussian Kernel, GK): Non-linear relationships are captured using a Gaussian kernel matrix. 4) *Model S4* (Additive Pedigree and Kernel Effects): The pedigree-based random effect (S1) and the Gaussian kernel (S3) are combined, allowing for additive effects. 5) *Model S5* (Symmetric Matrices ***C*** and ***P***): This model extends model S4 by incorporating two additional random effects with covariance matrices, ***C*** and ***P***, based on weighting between the additive relationship matrix derived from pedigree and kernel matrices. These matrices capture complex genomic relationships and are scaled to ensure proper variance components.

We extend these models to five multi-environment models with genotype-by-environment interactions (GE), namely: 1) *Model M1* (Pedigree-Based GE) uses a multivariate normal distribution with a ***U*** ⊗ ***A*** covariance matrix, where ***U*** is the covariance between environments, ***A*** is the pedigree matrix and ⊗ denotes the Kronecker product. 2) *Model M2* (GBLUP-Based GE) uses the GBLUP method, modeling linear relationships with a ***U*** ⊗ ***G*** covariance structure. In cases of unbalanced data, an alternative covariance matrix is introduced. 3) *Model M3* (Gaussian Kernel GE) incorporates non-linear marker relationships via the Gaussian kernel, modeling the GE interaction using ***U*** ⊗ ***K*** for balanced data or a custom covariance matrix for unbalanced data. 4) *Model M4* (Additive Pedigree and Kernel Effects GE) adds both pedigree and Gaussian kernel effects to the GE interaction model. 5) *Model M5* (Additive Effects with Symmetric ***C*** and ***P*** Matrices) expands model M4 by incorporating the ***C*** and ***P*** matrices, which capture additional covariance structures beyond those modeled by pedigree and kernel matrices. These models aim to improve predictive accuracy by leveraging both molecular marker information and pedigree data, with some incorporating complex genomic relationships through Gaussian kernels and custom covariance matrices.

## 2 Materials and methods

### 2.1 Phenotypic and genotypic data

Details of the phenotypic data used in this study are provided in **Table A1** (see Appendix). Within the International Center for Maize and Wheat Improvement (CIMMYT), The Elite Yield Trials (EYTs) are trials established in the wheat experimental station in Cd. Obregon, Mexico and planted every year under Alpha-Lattice design with two replicates in several simulated environments. The environments are B2IR=bed planting two irrigations, B5IR=bed planting five irrigations, BEHT=bed planting early heat trials, BLHT=bed planting late heat trials, and F5IR=flat planting five irrigation.

All genomic prediction analyses were conducted using a two-stage modeling framework. Trials were organized in multiple sub-trials or lattices sharing a variable number of checks. In the first stage, adjusted means or Best Linear Unbiased Estimates (BLUEs) were calculated using linear mixed models (LMMs) implemented in the ASReml-R package (https://vsni.co.uk/software/asreml-r/). The model accounted for the various lattice effects, with replication nested within lattice, and incomplete blocks nested within replication and lattice, all treated as random effects. Genotype factor was included as a fixed effect. Detailed model specifications are provided in Vitale et al. (2025). The resulting BLUEs were then used as response variables in the second stage for genomic prediction modeling.

Wheat DNA was isolated from dried leaf tissue collected from five representative plants per genotype using a modified cetyltrimethylammonium bromide (CTAB) extraction protocol. Genotyping was conducted via the Genotyping-by-Sequencing (GBS) approach on an Illumina HiSeq2500 platform at Kansas State University, following the methodology described by Poland et al. (2012). Raw sequence data underwent stringent quality control using TASSEL version 5.0 (https://tassel.bitbucket.io). The resulting genotypic dataset comprised 18,239 high-quality single nucleotide polymorphism (SNP) markers. SNPs with a minor allele frequency (MAF) below 5% or with over 70% missing data were excluded. Remaining missing genotypes were imputed using BEAGLE v5.0 to enhance data completeness. Post-processing, the filtered HapMap file was converted into a numerical genotype matrix to enable downstream genomic prediction analyses.

### 2.2 Genomic prediction single-environment models

A sequence of 11 equations (Equations 1–11) are described in the following sub-sections.

#### 2.2.1 Single-environment model S1

This semiparametric regression model includes random genomic effects with a covariance structure based on the pedigree, such as Crossa et al. (2010):


(1) $$y=1\;\mu +g_{A}+\epsilon$$

where the vector of phenotypic responses y is sized *n*× ***1***, vector 1 is also sized *n*×1 and *μ* is the intercept. The random vector $g_{A}$ follows a multivariate normal distribution $g_{A}\sim N\left(0,\sigma_{g_{A}}^{2}Z_{g}AZ_{g}^{'}\right)\;$ where $\sigma_{g_{A}}^{2}\;$ is the variance component and $Z_{g}$ is a matrix that relates the phenotypes to the relationship matrix derived from pedigree A . It is assumed that the vector of random errors follows a multivariate normal distribution with zero mean and homogeneous variance ϵ~N(0, $\sigma_{\epsilon }^{2}I)$ .

#### 2.2.2 Single-environment model S2

In this model, (similar to Equation 1) rather than using the pedigree relationship matrix, the linear genomic relationship matrix is used, computed as suggested by VanRaden (2008), although adjusted as in López-Cruz et al. (2015), taking advantage of the fact that the marker matrix is standardized as $G=XX^{'}/p$ , where p represents the number of markers.


(2) $$y=1\;\mu +g_{g}+\epsilon$$

where the random vector $g_{g}\sim N\left(0,\sigma_{g_{g}}^{2}Z_{g}GZ_{g}^{'}\right)\;$ follows a normal distribution and the other components are as in model S1.

#### 2.2.3 Single-environment model S3

In this model, (similar to Equation 2) genomic matrix K is known as the Gaussian kernel (GK), representing non-linear relationships and constructed as in Crossa et al. (2010)


(3) $$y=1\;\mu +g_{K}+\epsilon$$

where the random vector $g_{K}$ has a normal distribution $g_{K}\sim \;N\left(0,\sigma_{g_{K}}^{2}Z_{g}KZ_{g}^{'}\right),$ with K constructed with the Euclidean distance d  between individuals measuring the markers (see Equation 4)


(4) $$K_{ij}=\exp\left(-\frac{d_{ij}^{2}}{median\left(d_{ij}^{2}\right)}\right)\text{ for}\;i=1,\ldots ,n\;;j=1,\ldots ,n$$

#### 2.2.4 Single-environment model S4

In this model, independent random vectors are considered within an additive model, with covariance structures such as those in models S1 and S3 (Equations 1, 3), as in Crossa et al. (2010).


(5) $$y=1\;\mu +g_{A}+g_{K}+\epsilon$$

#### 2.2.5 Single-environment model S5 (proposed model)

In the previous model (Equation 5), the relationship matrices from the pedigree A and the Gaussian kernel K , constructed using the markers, were used as covariance matrices for two independent random effect vectors. In the current model, two additional independent random vectors are added, using two symmetric matrices that we will call C and P as covariance matrices. These matrices ( C and P ) are derived from the multiplication of K and A .

Thus, the proposed model is


(6) $$y=1\;\mu +g_{A}+g_{K}+g_{C}+g_{P}+\epsilon$$

where random vectors $g_{C}\text{ and }g_{P}$ are added to model S4 with the aim of capturing effects that were not previously included. The additional random vectors follow normal distributions $g_{C}\sim \;N\left(0,\sigma_{g_{C}}^{2}Z_{g}CZ_{g}^{'}\right)$ and $g_{P}\sim \;N\left(0,\sigma_{g_{P}}^{2}Z_{g}PZ_{g}^{'}\right)$ .

S5 (Equation 6) is an extension of the S4 (Equation 5) genomic prediction model that incorporates two additional covariance matrices, ***C*** and ***P***, derived from the multiplication between the genomic relationship matrix (***K***) and the pedigree relationship matrix (***A***). These matrices capture an additional genetic variance not fully explained by ***A*** and ***K*** alone.

In summary, model S5 improved prediction accuracy by integrating two novel symmetric kernels, ***C*** and ***P*** derived from the upper and lower triangular parts of the product between the genomic and pedigree matrices. These additional covariance structures captured independent genetic signals, enhancing the model’s ability to explain complex genetic relationships beyond those captured by standard genomic or pedigree-based approaches.

### 2.3 Genomic prediction multi-environment models

#### 2.3.1 Multi-environment model M1

Wheat breeding programs typically involve testing across multiple environments, making it crucial to account for genotype-by-environment (G×E) interactions in prediction models. Properly modeling these interactions can substantially improve prediction accuracy. In this context, we evaluated whether incorporating both pedigree (***A*** matrix) and genomic (***K*** matrix) information could also enhance model performance under a multi-environment framework. All models described below explicitly account for G×E interactions.

The first model (M1) includes only the pedigree-based relationship matrix (***A***).


(7) $$y=\mu +g_{A\;}+\epsilon$$

where $y={\left(y_{.1},\ldots y_{.m.}\right)}^{'}$ and $y_{.1}$ sized $n_{1}$ for each of the m environments, such that n represents the total number of cultivars in all environments. Similarly, for each component, $\mu ={\left(\mu_{.1},\ldots \mu_{.m.}\right)}^{'}$ where $\mu_{.j}=1_{j}\mu_{j}$ for j=1,…,m .

When the data are balanced and genotypes are arranged within each environment, the vector of additive genetic effects across all environments $g_{A\;}$ follows a multivariate normal distribution with a mean of zero and $U_{A}⊗A$ covariance matrix (Cuevas et al., 2017), where $U_{A}$ can be an unstructured covariance matrix between the environments, which will be estimated and is sized *m×m*, and A is the pedigree matrix, and the symbol ⊗ denotes the Kronecker product. In the case of random errors, they also follow a multivariate normal distribution with a mean of zero, but with covariance matrix R⊗I , where R is a covariance matrix of random errors between the environments to be estimated of an *m×m* order and ***I*** is the identity matrix Note that Martini et al. (2020) showed that the covariance matrix $U_{A}⊗A$ can be expressed as $Z_{1}AZ_{1}^{'}\circ Z_{2}EZ_{2}^{'}$ with appropriate design matrices $Z_{1}$ and $Z_{2}$ , which relate phenotypes to the respective genotype by environment combination, and where E is the covariance matrix between the environments, and where ∘  denotes the Hadamard product.

In cases where not all genotypes were tested in each environment, the covariance matrix of $g_{A\;}$ was modelled as $\sigma_{g_{A\;}}^{2}Z_{1}AZ_{1}^{'}\circ Z_{2}EZ_{2\;}^{'}$ .

#### 2.3.2 Multi-environment model M2

This model only considers the linear relations between markers, that is, GBLUP


(8) $$y=\mu +g_{G\;}+\epsilon$$

The difference with model M1 (Equation 7) is using markers instead of pedigree to establish the genome relationship between cultivars. When data is balanced, it is assumed that $g_{G\;}$ has a Multivariate Normal distribution with a mean of zero and a $U_{g}⊗G$ variance covariance. When data is unbalanced, the variance covariance matrix to be used is $\sigma_{g_{g}}^{2}Z_{1}GZ_{1}^{'}\circ Z_{2}EZ_{2}^{'}$ , as pointed out in the last section.

#### 2.3.3 Multi-environment (model M3)

This model considers the marker relationship as the non-additive Gaussian kernel K .


(9) $$y=\mu +g_{K\;}+\epsilon$$

As in other cases, if data is balanced, $G_{K\;}$ has a variance covariance matrix $U_{K}⊗K$ . Otherwise, if data was unbalanced, the variance covariance would be $\sigma_{g_{K}}^{2}Z_{1}KZ_{1}^{'}\circ Z_{2}EZ_{2}^{'}$ with the covariance of the error, as in the previous case.

#### 2.3.4 Multi-environment (model M4)

This model considers the summation of $g_{A\;}\text{and }\;g_{K\;}$


(10) $$y=\mu +g_{A\;}+g_{K\;}+\epsilon$$

The components are the same as in models M1 (Equation 7) and M3 (Equation 9).

#### 2.3.5 Multi-environment model M5 (proposed model)

This model adds $g_{C\;},\;g_{P\;}$ to the previous model (Equation 10):


(11) $$y=\mu +g_{A\;}+g_{K\;}+g_{C}+\;g_{p}+\epsilon$$

where $g_{C\;},\;g_{P\;}$ assumes multivariate normal distribution with a mean of zero and covariances $U_{C}⊗C$ , $U_{P}⊗P$ , for balanced data with C,P as in model M5.

For unbalanced data, the covariance matrices of $g_{C\;}$ and $g_{P\;}$ are modelled as $\sigma_{g_{C}}^{2}Z_{1}CZ_{1}^{'}\circ Z_{2}EZ_{2}^{'}$ or $\sigma_{g_{p}}^{2}Z_{1}PZ_{1}^{'}\circ Z_{2}EZ_{2}^{'}$ , respectively.

In brief, model M5 (Equation 11) extended the hybrid kernel approach to multi-environment genomic prediction by incorporating genotype-by-environment interactions alongside the novel ***C*** and ***P*** kernels. This integration allowed M5 to capture both complex genetic relationships and their environment-specific expressions, resulting in improved predictive performance across diverse environments.

### 2.4 Construction and rationale of symmetric kernal *C* and *P* from *K* and *A*

Here we present a comprehensive summary of the matrix operations employed. **Table 1** distinguishes between different types of matrix products—dot, Hadamard, and Kronecker—used to model various components of genetic and genotype-by-environment (G×E) interactions. Each operation serves a distinct purpose: Kronecker products (e.g., ***U*** ⊗ ***A***) are used to model G×E covariance structures, dot products (e.g., ***K*** × ***A***) to derive hybrid kernels, and Hadamard products (e.g., ***K*** ∘ ***A*** to combine information elementwise. By clearly specifying the role, interpretation, and appropriate usage of each matrix expression, this table provides a concise yet detailed guide for readers navigating the statistical modeling framework. Finaly an R code was provided in **Supplementary Materials Box 1**.

**Table 1:** Interpretation of matrix operations used in genomic prediction models.

Matrix expression	Component roles	interpretation	Usage recommendation
**U** ⊗ A*	**U**: Environment covariance **A**: Pedigree matrix	Captures G×E interaction by combining environment and pedigree structures through Kronecker product.	Standard for modeling G×E with pedigree-based relationships.
**U** ⊗ **K**	**U**: Environment covariance **K**: Genomic matrix (kernel)	Captures G×E interaction with realized genomic relationships across environments.	Recommended for G×E modeling with genomic data.
**K** ⊗ **A**	**K**: Genomic kernel **A**: Pedigree matrix	Combines two genetic structures; not interpretable for G×E modeling.	Avoid in G×E modeling.
**K** × **A**	**K**: Genomic kernel **A**: Pedigree matrix	Matrix (dot) product producing a new kernel. Not symmetric; used to derive kernels **C** and **P**.	Used to construct hybrid kernels **C** and **P**.
**K** ∘ **A**	**K**: Genomic kernel **A**: Pedigree matrix	Hadamard (element-wise) product. Each element is the product of corresponding elements from **K** and **A**.	Can be used when combining kernels pointwise; less common than **K** × **A**.

*Bold values indicate matrices annotation.

#### 2.4.1 Description of *K, A*, and the matrix *KA* product

Let ***K*** and ***A*** be two square matrices of the same dimension n × n, where each element (i,j) represents the genetic relationship between genotype i and genotype j. Matrix ***K*** is a genomic relationship matrix computed from molecular marker data, while matrix ***A*** is a pedigree-based relationship matrix.

The matrix product ***KA*** = ***K × A*** yields a non-symmetric matrix, where each element in position (i,j) represents the scalar (dot) product between the i-th row of ***K*** and the j-th column of ***A***. Conceptually, this measures how the genomic relationships of genotype i align with the pedigree-based relationships of genotype j. Thus, the ***KA*** matrix reflects the projection of the genomic profile of genotype i onto the pedigree structure of genotype j.

From the matrix ***KA***, the diagonal elements are extracted and averaged. Next, the matrix is split into its upper triangular and lower triangular parts. The upper triangular part (UT) includes the elements above the main diagonal, while the lower triangular part (LT) includes the elements below the main diagonal.

#### 2.4.2 Construction of symmetric kernels *C* and *P*

Two new symmetric matrices are then constructed. Matrix ***C*** *i*s created by summing the upper triangular part of ***KA*** and its transpose, ensuring symmetry. Its diagonal is replaced with the original diagonal of ***KA***, and the resulting matrix is scaled by dividing all entries by the average of its diagonal values.

Similarly, matrix ***P*** is constructed by summing the lower triangular part of ***KA*** and its transpose. The diagonal is again replaced with that of ***KA***, and the matrix is scaled by the average of its diagonal values. This process ensures that both ***C*** and ***P*** retain the diagonal of the original matrix ***KA*** while achieving symmetry and proper scaling.

#### 2.4.3 Statistical interpretation, implications and justification for *C* and *P*

Although ***KA*** is derived from valid kernels ***K*** and ***A***, it is not symmetric and therefore not itself a valid kernel. A naive symmetrization of ***KA*** (1) does not improve prediction, (2) it may satisfy symmetry but not necessarily improve or preserve positive semi-definiteness, biological meaning, or predictive accuracy, (3) it is a default or simplistic approach without a theoretical basis in the context of kernel methods.

In contrast, constructing matrices ***C*** and ***P*** from the upper and lower triangular parts of ***KA*** provides valid kernel matrices. These satisfy the three essential conditions for valid kernels: (1) symmetry, (2) positive semi-definiteness (PSD), and (3) Mercer’s condition for continuous kernels.

However, although matrices ***C*** and ***P*** are both derived from ***KA***, they capture different and complementary aspects of the genetic relationship structure. This is evidenced by their distinct variance components and the low correlation between the genomic predictions obtained from ***C*** and ***P*** compared to those from ***K*** or ***A***. As such, ***C*** and ***P*** are not redundant but instead provide independent genetic signals that can enhance the accuracy of genomic effect predictions when used in combination with other kernels.

### 2.5 Implementation of predictions

Models were fitted using the BGLR package (Pérez and de los Campos, 2014), utilizing the BGLR function for Single Environment models (S1,…,S5) and the Multi-trait function (Pérez-Rodríguez and de los Campos, 2022) for multi-environment models (M1,…,M5). Inferences were based on 50,000 MCMC (Markov Chain Monte Carlo) iterations, 5000 burning and 5 thinning.

### 2.6 Cross-validations

Using the phenotypic data, a training set and a testing set were formed for model evaluation. The training set is used with phenotypic data to predict the phenotypic data of the test set and the predictive ability is measured by comparing the predicted values with the true values. For each data group and environment, Pearson’s correlation coefficient between the predicted data and the true values was calculated.

**Figure 1:**
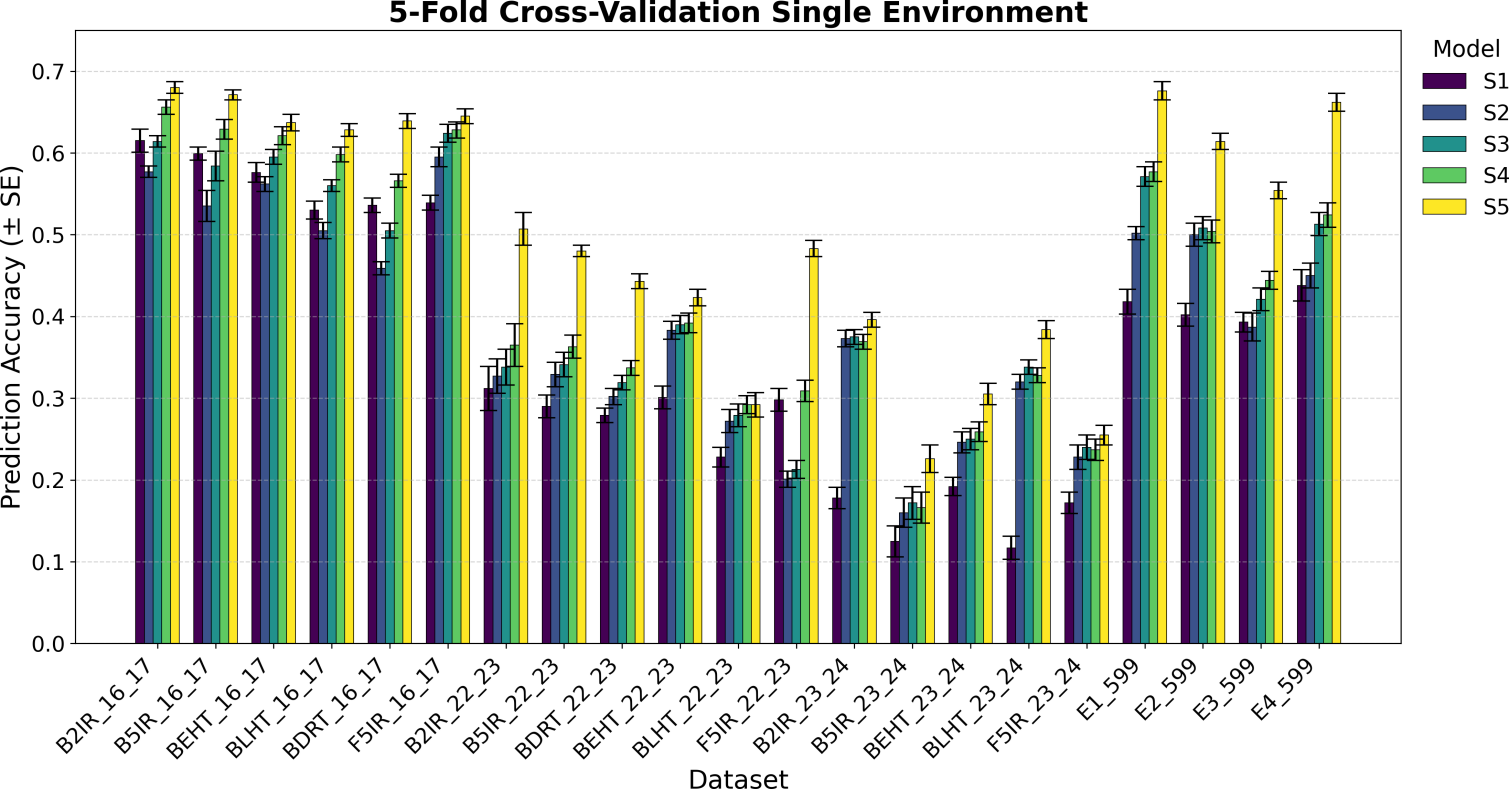
Average Pearson’s correlation coefficients (AVG) and corresponding standard errors (SE) across four cycles of 5-fold cross-validation for single-environment genomic prediction models of grain yield. Results are shown for the following datasets: EYT_16_17 (B2IR, B5IR, BEHT, BLHT, BDRT, and F5IR), EYT_22_23 (B2IR, B5IR, BEHT, BLHT, BDRT, and F5IR), EYT_23_24 (B2IR, B5IR, BEHT, BLHT, and F5IR), and Wheat 599 (E1, E2, E3, and E4).

**Figure 2:**
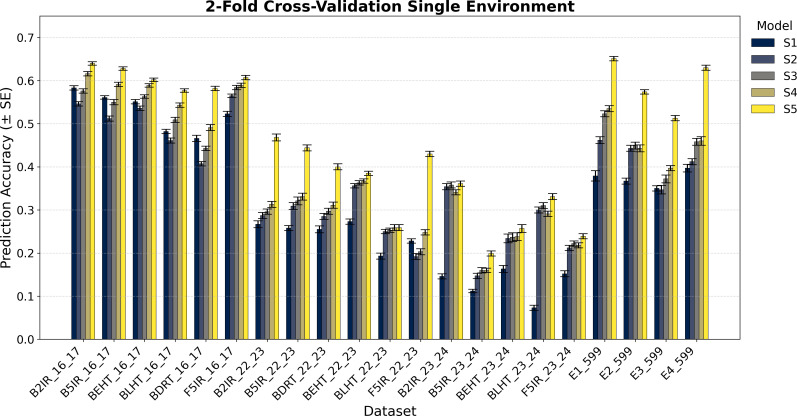
Average Pearson’s correlation coefficients (AVG) and corresponding standard errors (SE) across ten cycles of 2-fold cross-validation for single-environment genomic prediction models of grain yield. Results are shown for the following datasets: EYT_16_17 (B2IR, B5IR, BEHT, BLHT, BDRT, and F5IR), EYT_22_23 (B2IR, B5IR, BEHT, BLHT, BDRT, and F5IR), EYT_23_24 (B2IR, B5IR, BEHT, BLHT, and F5IR), and Wheat 599 (E1, E2, E3, and E4).

**Figure 3:**
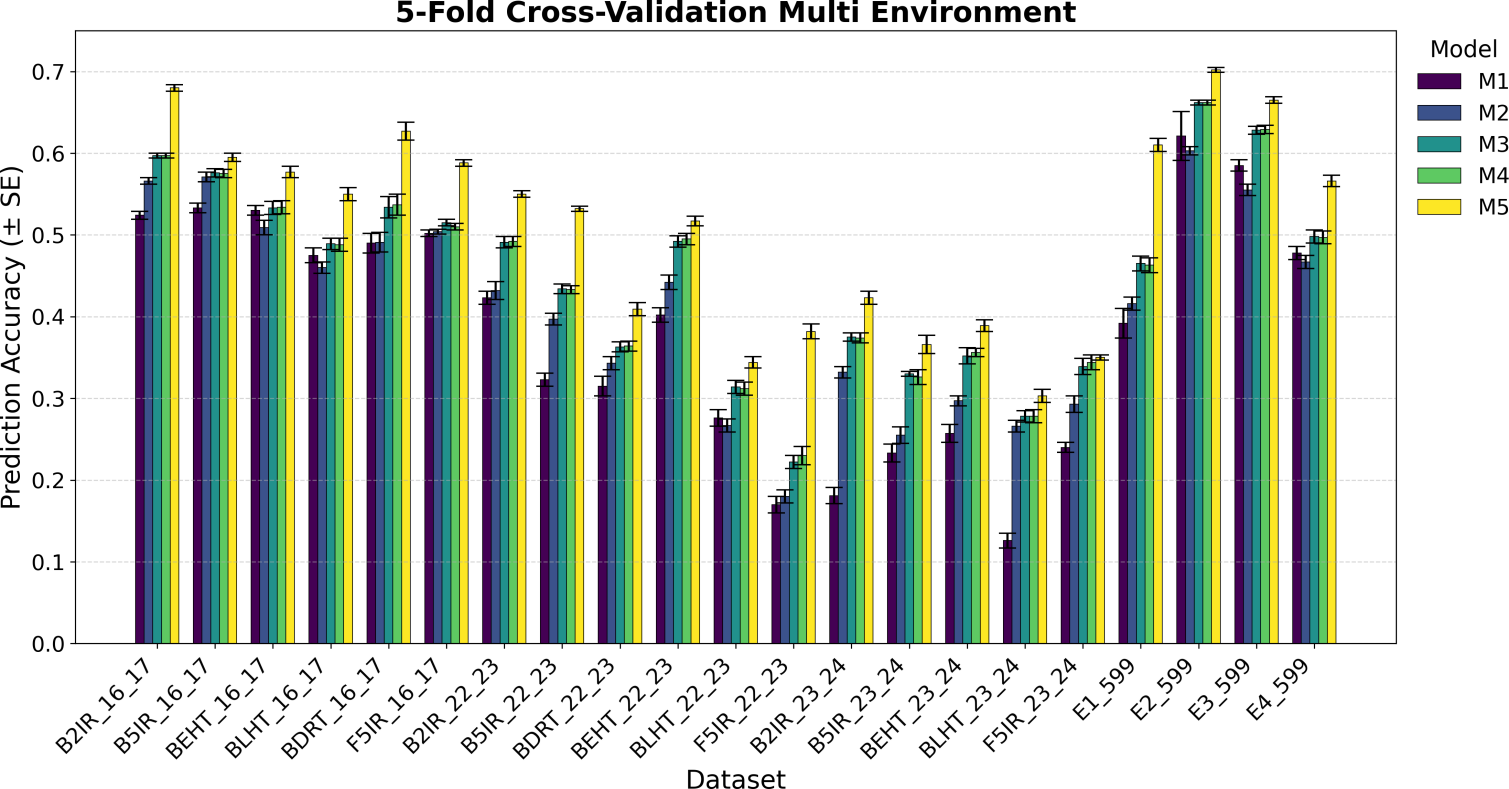
Average Pearson’s correlation coefficients (AVG) and corresponding standard errors (SE) across four cycles of 5-fold cross-validation for multi-environment genomic prediction models of grain yield. Results are shown for the following datasets: EYT_16_17 (B2IR, B5IR, BEHT, BLHT, BDRT, and F5IR), EYT_22_23 (B2IR, B5IR, BEHT, BLHT, BDRT, and F5IR), EYT_23_24 (B2IR, B5IR, BEHT, BLHT, and F5IR), and Wheat 599 (E1, E2, E3, and E4).

**Figure 4:**
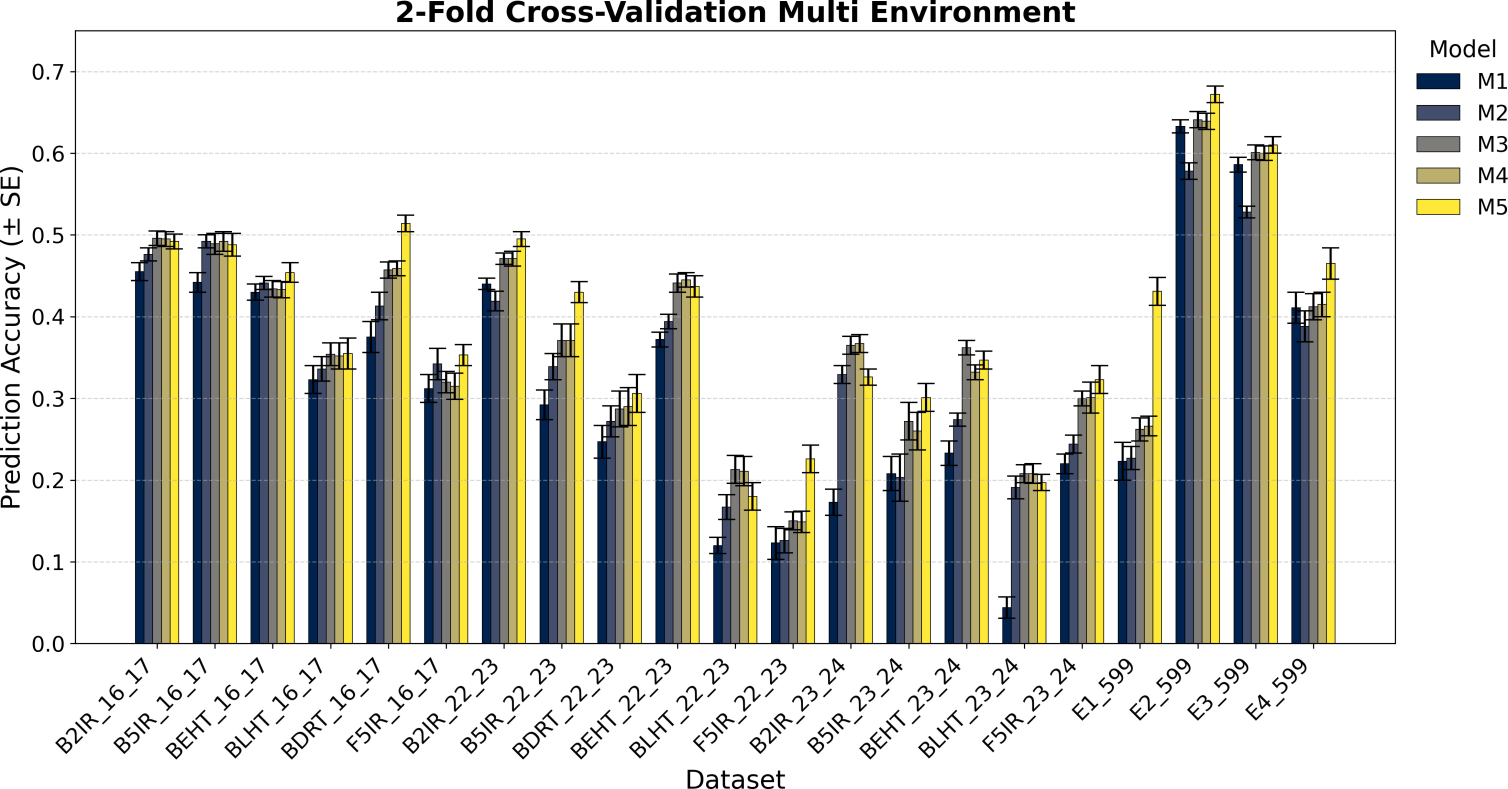
Average Pearson’s correlation coefficients (AVG) and corresponding standard errors (SE) across ten cycles of 2-fold cross-validation for multi-environment genomic prediction models of grain yield. Results are shown for the following datasets: EYT_16_17 (B2IR, B5IR, BEHT, BLHT, BDRT, and F5IR), EYT_22_23 (B2IR, B5IR, BEHT, BLHT, BDRT, and F5IR), EYT_23_24 (B2IR, B5IR, BEHT, BLHT, and F5IR), and Wheat 599 (E1, E2, E3, and E4).

The variance component estimation of each data set EYT 16_17, EYT 22-23, EYT 24-25, and Wheat 599 is in **Table 2**, whereas the averages of the predictive correlations (AVG) and their corresponding standard errors (SE) are reported in **Figures 1**–**4**. **Table 3** in the section of Conclusion summarizes the results for all models These measures are complemented by calculating the average of the squared predictive errors (PMSE) to verify that a higher predictive correlation corresponds to a lower PMSE.

**Table 2:** Variance components of model S5 for the four data sets EYT_16_17 (B2IR, B5IR, BEHT, BLHT, BDRT, and F5IR) EYT_22_23 (B2IR, B5IR, BEHT, BLHT, BDRT, and F5IR), EYT_23_24 (B2IR, B5IR, BEHT, BLHT, and F5IR), and Wheat 599 (E1, E2, E3, and E4).

Dataset	$\sigma_{g_{A}}^{2}$	$\sigma_{g_{K}}^{2}$	$\sigma_{g_{C}}^{2}$	$\sigma_{g_{P}}^{2}$	$\sigma_{\epsilon }^{2}$
B2IR_16_17	0.199	0.449	0.344	0.266	0.215
B5IR_16_17	0.115	0.455	0.801	0.620	0.186
BEHT_16_17	0.184	0.587	0.111	0.280	0.230
BLTH_16_17	0.177	0.721	0.469	0.228	0.156
DRT_16_17	0.253	0.336	0.571	0.436	0.235
F5IR_16_17	0.085	0.809	0.120	0.124	0.204
B2IR_22_23	0.056	0.365	0.585	0.463	0.386
B5IR_22_23	0.078	0.360	0.357	0.202	0.445
BDRT_22_23	0.064	0.362	0.292	0.235	0.485
BEHT_22_23	0.072	0.576	0.183	0.153	0.415
BLHT_22_23	0.069	0.573	0.098	0.068	0.534
F5IR_22_23	0.071	0.352	0.533	0.501	0.400
B2IR_23_24	0.050	0.592	0.112	0.152	0.470
B5IR_23_24	0.052	0.242	0.114	0.173	0.690
BEHT_23_24	0.059	0.485	0.180	0.105	0.551
BLHT_23_24	0.050	0.650	0.189	0.111	0.437
F5IR_23_24	0.071	0.394	0.103	0.082	0.633
E1	0.061	0.350	0.188	0.241	0.256
E2	0.048	0.227	0.259	0.189	0.403
E3	0.076	0.282	0.202	0.260	0.383
E4	0.069	0.288	0.209	0.184	0.312

**Table 3:** Comparative Summary of Models S1–S5 and M1–M5.

Model	Description	Advantages	Limitations/recommended use	Mean prediction performance (Pearson’s)
S1	Pedigree-based model using matrix A* only.	Useful when marker data is unavailable; captures expected genetic relationships.	Limited accuracy; does not account for realized genetic variation. Suitable for basic predictions.	~ 0.30
S2	GBLUP model using genomic **matrix** G (linear).	Incorporates marker data; better than S1 for realized additive effects.	Fails to model non-linear effects; assumes constant variance for all markers.	~0.35
S3	Gaussian kernel model (non-linear genomic relationships).	Captures epistasis and complex genetic interactions via non-linear structure.	More computationally intensive; requires dense, high-quality marker data.	~0.38
S4	Additive model combining **A** and **K** as independent effects.	Balances pedigree and genomic data; improves prediction over S1–S3.	Still assumes additivity; may not fully exploit marker–pedigree interaction.	~0.41
S5	Proposed hybrid model using kernels **C** and **P** derived from **K × A**.	Captures additional independent genetic variance; consistently best prediction in SE models.	Requires computation of C and P; best used when both A and K are available.	~0.50–0.68
M1	Multi-environment model using **U** ⊗ **A.**	Models G×E using pedigree data; interpretable and simple.	Not suitable for complex genomic relationships; limited precision without marker data.	~0.40
M2	GBLUP-based G×E model using **U** ⊗ **G**.	Leverages genomic data for G×E; more precise than M1.	Assumes linear relationships; may miss epistasis.	~0.45
M3	Gaussian kernel G×E model using **U** ⊗ **K.**	Captures non-linear G×E patterns; ideal for complex environments.	High computational demand; sensitive to kernel bandwidth.	~0.48
M4	Additive G×E model with both **A** and **K**.	Combines pedigree and genomic information for G×E modeling.	Still assumes additivity; may underfit complex interactions.	~0.50
M5	Proposed hybrid G×E model with kernels C and P.	Captures independent and complex G×E variance; top performer in most cases.	Most demanding in terms of data and computation; best when rich phenotypic, A, and K data are available.	~0.55–0.70

*Bold values indicate matrices annotation.

#### 2.6.1 5-fold and 2-fold cross-validations

For the single-environment models, we employed two cross-validation schemes to evaluate prediction accuracy. In the first scheme, a 5-fold cross-validation was conducted, in which the dataset was randomly divided into five equal parts. In each iteration, four parts were used for training and one part (i.e., 20% of the data) was used for testing. This process was repeated four times so that each observation was included in the test set once. To assess the stability of the results, the entire 5-fold cross-validation procedure was repeated four times, yielding a total of 20 independent training-testing combinations, each with 20% of the data being masked and predicted.

In the second scheme, we applied a more stringent 2-fold cross-validation, where the dataset was randomly split into two equal halves. In each iteration, 50% of the data was masked for testing, and the remaining 50% was used for training. This approach simulates more challenging prediction scenarios with a larger proportion of missing data. To ensure robustness, the 2-fold procedure was repeated ten times, resulting in 20 different training-testing combinations, each with 50% of the data masked.

The prediction results for the five single-environment models (S1–S5) are summarized in **Figures 1** and **2**, and for the five multi-environment models (M1–M5) in **Figures 3** and **4**.

## 3 Results organization

As previously mentioned, the main presentation and description of the results are given in **Figures 1–** **4**. Appendix contains several tables, **Supplementary Table S1** gives detailed information of the environment for each of the 5 data sets; **Supplementary Tables S2** and **Supplementary Table S3** provide correlations between the predicted vectors of models S5 on data EYT-16_17 and EYT-22_23 (**Supplementary Table S2**) and EYT-23_24 and Wheat 599 (**Supplementary Table S3**). **Supplementary Table S4** shows the average and SD of PMSE for single-environment models for trials EYT16_17, and EYT 22_23 are shown for 4 repetitions with five folds and 10 repetitions for two folds. Also shown is the difference as a percentage of the predictive averages between the proposals (S5) and the GBLUP model (S2). Similar is shown for **Supplementary Table S5** but for datasets EYT_23–24 and for data Wheat 599. Finally, **Table S6** and **Table S7** have the average and SD of PMSE for EYT_16_17 and EYT _22_23, EYT_23_24 and Wheat 599, in different environments and the 5 multi-environment (M) models. Two cases are shown: five- and two-fold cross-validations. Also shown is the difference as a percentage of the predictive averages between the proposals (M5) and the GBLUP model (M2).

### 3.1 Results

**Table 2** shows the variance components of the random vectors from model S5 for each of environments included in each of the 5 data sets, for which values of the vector of y observations were standardized. It can be noticed that the contribution of the variance components $\sigma_{g_{C}}^{2}\;,\;\sigma_{g_{P}}^{2}$ are important and as their values increase, so does the predictive ability if compared with models S1, S2, S2 and S4, as shown by **Figures 1** and **2**.

The table in Appendix S2 and Appendix S3 shows that the correlations of the predicted vectors $g_{C},g_{P}$ with vectors $g_{A},g_{K}$ are low, indicating little or no association, that is, the information is not duplicated and the components $g_{C},g_{P}$ provide additional information.

**Figures 1** and **2** show the results for the data groups **EYT_16_17 and EYT_22_23**. In addition to the averages and standard errors of the predictive performance using Pearson´s correlations, the percentage differences of the proposed model (S5) against the GBLUP model (S2) are reported. The highest averages in terms of Pearson´s correlations (marked in bold) correspond to S5, in both five-folds and two-folds for both datasets. For the **EYT_16_17** dataset, the average, in terms of Pearson´s correlations, is highest in environment B2IR_16_17 for S5, with a value of 0.680, while the greatest percentage difference between S5 and S2 is found in BDRT_16_17, with 43% for two-folds. Conversely, the smallest difference is in environment F5IR_16_17, with 7% in two folds. If we construct 95% confidence intervals by adding and subtracting the result of multiplying the standard error by 1.96 from the mean, we could observe that the intervals of S5 and S2 do not overlap, indicating that their differences are significant. We can also observe that the models ordered from the highest to lowest correlation averages are S5, S4, S3, S1, and S2 in both five-fold and two-fold cases. It is interesting to note that the S1 model with a compound covariance matrix with pedigree yields better predictions than model S2 (GBLUP). When comparing S5 with the next best model (S4), the differences remain significant, and their confidence intervals do not overlap.

For the **F5IR_22_23** dataset, the model with the highest average in terms or Pearson´s correlations is, once again, S5 in both five-fold and two-folds. The environment with the highest average Pearson´s correlation is B2IR_22_23, with 0.507 for five-folds, whereas the greatest percentage difference between S5 and S2 is in environment F5IR_22_23 with 140%, and the smallest difference corresponds to environment BLHT_22_23 with 4% for two-folds. If confidence intervals were constructed using the averages of these two models and their standard errors, the intervals would not overlap, indicating a good level of significance, except for BLHT_22_23. The models ordered from highest to lowest predictive ability are S5, S4, S3, S2, and S1. In this dataset, model S2 has a higher average correlation than model S1, unlike the previous dataset. When comparing models S5 and S4, the differences decrease but remain noteworthy. In both data groups, the averages of Pearson’s correlations decrease when predicting 50% of missing data (two-folds) compared to predictions of 20% (five-folds). However, the percentage differences between the models remain within an acceptable range.

**Figures 1** and **2** also show the average Pearson’s correlations and standard errors for the data sets **EYT_23_24** and Wheat 599 data groups, along with the percentage differences between models S5 and S2 for the five-fold and two-fold cases. The best predictive model for the datasets is model S5. For EYT_23_24, the environment with the highest predictive average is B2IR_23_24, with a value of 0.396. In contrast, this environment has the smallest percentage difference for five-folds and two-folds, with 6% and 2%, respectively. The lowest average for S5 is 0.199 for environment B5IR_23_24, yet it also has the largest percentage difference between S5 and S2 with 41% and 36% for five-folds and two-folds. Unlike the previous data groups, S3 has a higher predictive ability than S4, and the differences between S3 and S2 are smaller. By observing the averages and standard errors, we can deduce that the differences between the averages of S5 and of the other models are significant. For the **Wheat 599** dataset, a similar behavior is observed, with S5 being the best predictive model, followed by S4, S3, S2, and S1. The percentage differences are significant between S5 and the other models, according to the average values and standard errors. The highest average is 0.676 for E1_Wheat 599 for five-folds, and the largest percentage difference between S5 and S2 corresponds to environment E4_Wheat 599 with 53% for two folds. The lowest average of correlations for S5 is 0.513 for E3_Wheat 599 for two-folds. This environment also has the lowest average—of 0.3—for model S1 in two-folds. As in the other datasets, when comparing the prediction of 50% of the data (two-folds) with the prediction of 20% (five-folds), predictive ability tends to decrease, but the percentage differences of S5 with the other models remain significant.

**Figures 3** and **4** display the averages of Pearson’s correlations (AVG) and standard errors (SE) under the multi-environment models (M), along with the percentage differences of the averages between M5 and M2 for the EYT_16_17 and EYT_22_23 datasets. In these multi-environment models, two scenarios were used. In the first, it was considered that the phenotypic values of 20% of an environment are known, and 80% of that environment is to be predicted, while the values of the other environments are also known. In the second scenario, 5% of the phenotypic values of an environment are known, and 95% of the environment is predicted, while the values of the other environments are also known. In each scenario, 10 random samples were used as test data. For EYT_16_17, the averages and percentage differences are observed to be higher by 80% than for 95%. Thus, for 80%, M5 has the highest averages for the six environments, whereas for 95%, it only has higher averages in the four environments. Considering the averages and standard errors, we can deduce that for the 80% prediction, the difference between the averages of M5 and the other models is significant, but this is not the case for the 95% prediction, where, except for DRT_16_17, the other environments are not significant. The highest average is 0.680 for environment B2IR_16_17, and the lowest average for M5 is 0.355. The highest percentage difference is 28% for the BDRT_16_17 and the lowest is -1%, corresponding to the B5IR_16_17 environment for the 95% prediction. For the EYT_22_23 data, similarly to the previous data, the highest averages correspond to M5 for the 80% prediction, but it is only higher in the four environments for the 95% prediction. Likewise, by observing the averages and standard errors, we can deduce that the differences between the averages of M5 and the other models are significant, as constructing confidence intervals for the mean with a distance of 1.96 × SE does not overlap for the 80% predictions, although this is not the case for all situations for the 95% predictions. In this dataset, the highest value is 0.551 for B2IR_22_23 and the lowest value for M5 is 0.180 for environment BLHT_22_23.

**Figures 3** and **4** also present the indicators for the EYT_23_24 and Wheat 599 datasets. In both datasets, model M5 has higher predictive averages for the problem of predicting 80% of the data. The EYT_23_24 dataset only has a higher predictive average in two environments for the 95% prediction case. In this data group, for the 80% case, the differences between the averages of M5 and the other models are significant in magnitude, with the maximum percentage difference between M5 and M2 being 43%, and the minimum, 14%. In the case of the 95% prediction, only in the environment F5IR_23_24 does model M5 show better predictive ability than the other models. In the Wheat 599 dataset, the results are different, with model M5 showing higher averages in both scenarios (80% and 95% predictions). The differences between models are also significant between M5 and the other models, except in E3_Wheat 599, where M5 does not have a notable difference with M4 and M3 in the 95% prediction scenario. The highest average in this dataset is 0.702, corresponding to environment E2_Wheat 599, and the lowest average for M5 is 0.431 for E1_wheat 599 in the 95% prediction scenario. By contrast, this is where the greatest percentage difference (90%) between M5 and M2 is observed.

## 4 Discussion

In genomic prediction, ***A*** and ***G*** are two essential matrices used to represent relationships across individuals in a population. The pedigree relationship matrix (***A***) represents the expected genetic relationships between individuals based on their known pedigree. It quantifies the proportion of alleles that are identical by descent (IBD) between pairs of individuals based on Mendelian inheritance from recorded ancestry. It is derived from the pedigree records and reflects the expected shared genetic material based on family relationships. It has traditionally been used in animal and plant breeding to estimate breeding values, and it helps account for relatedness between individuals when predicting phenotypes (Legarra et al., 2009; Crossa et al., 2017).

Matrix ***A*** reflects broad, expected relationships but does not capture fine-scale variation in the way in which genes are inherited within a family. For example, full siblings are assumed to share 50% of their genes, but they might share slightly more or less, due to random Mendelian segregation.

The genomic relationship matrix (***G***) is based on molecular marker data (e.g., SNPs) and can capture the realized identity by state (IBS) accounting for the actual recombination, or mutations. Unlike the pedigree matrix, which is based on expected relationships, the genomic matrix is based on the actual shared genetic material observed through genotyping. The ***G*** matrix has become more accurate in genomic prediction, since it reflects the true genetic similarities between individuals, capturing the variation in inheritance that may not be evident from pedigree alone.

To calculate the ***G*** matrix, the product ***XX’*** is used, where the relationship between each genotype can be seen as the number of marker pairs with prevalence (Meuwissen et al., 2017), assuming their values are classified as 1, 0 (Slater et al., 2016). Furthermore, the ***K*** matrix captures the additive effects of the markers (as with the ***G*** matrix), as well as other nonlinear or complex effects such as epistasis, which are present in wheat.

### 4.1 Combining matrices, *A* and *K*

Although it is possible to combine the ***G*** and ***A*** matrices as independent random vectors (Crossa et al., 2010; Howard et al., 2019), in this work, the Gaussian kernel (***K***) was used as the genomic matrix in the additive combination of independent vectors with covariance matrices, as in models S4 and M4, since it generally produces higher predictions, allowing for comparisons with the proposed methods.

Although matrix ***A*** (pedigree relationship matrix) and matrix ***K*** (genomic relationship matrix) are calculated using different data sources (pedigree *vs*. genomic markers), they are indeed related because they both aim to capture genetic relationships between individuals.

While the variance-covariance matrix ***K*** is based on the (exponentiation of) squared distances between two genotypes, these squared distances can be viewed as the number of differing markers between two genotypes if we assume their values are 1 or 0 (prevalence) (Meuwissen et al., 2017; Slater et al., 2016). By scaling these distances with a bandwidth parameter *h*, we can broaden or narrow the covariance spectrum and then smoothen the result using a negative exponential function to capture complex effects. In contrast, matrix ***A*** represents the kinship relationship between genotypes or lines, generally expressed as a proportion.

In some genomic prediction strategies, combining matrices ***A*** and ***K*** can enhance prediction accuracy. The strategy used in this study was to combine information from matrices ***K*** and ***A*** by applying matrix multiplication to both matrices. The result is a restructured matrix in which each element represents the weighting of each row of ***K*** with the columns of ***A*** (generally proportions). This approach incorporates both marker and pedigree information when assessing the relationship between genotypes. Geometrically, this can be interpreted as projecting one vector onto another, akin to the convolution of two functions with the aim of smoothing the result. This principle is also used in deep learning convolutional neural networks as a core component (Montesinos-López et al., 2022)

However, the resulting matrix, KA,  is not symmetric and cannot be proposed as a covariance matrix. To address this, the upper triangular part is assigned to both the upper and lower triangular parts of a new matrix called ***C***. Similarly, the lower triangular part of the point product ***KA*** is assigned to both the upper and lower triangular parts of a new matrix called ***P***. Note that the lower triangular part of ***KA*** is equal to the upper triangular part of ***AK***. This method creates two symmetric covariance matrices. Both matrices ***C*** and ***P*** have the diagonal of ***KA*** and are scaled by dividing them by the means of their diagonal. Matrix ***C*** can be interpreted as the weighting of ***K*** with ***A***, while matrix ***P*** represents the weighting of ***A*** with ***K***. This hybrid matrix can be valuable in capturing both the expected (pedigree) and realized (genomic) genetic relationships, providing a comprehensive representation of the population’s genetic structure.

Combining matrices, ***A*** and ***K*** brings together the expected relationships from pedigree data with the more precise information from genomic data. With this combination, we have observed an empirical improvement of prediction accuracy in the wheat datasets because it captures both the coarse and fine genetic structure of the population.

In this study, by combining matrix ***K*** with matrix ***A*** (and vice versa), we are creating a hybrid relationship matrix that merges the strengths of both genomic and pedigree data in a novel way.

This combination leverages the fine-scale genetic variation captured by genotyping (***K***) for some pairwise comparisons while still incorporating the expected pedigree relationships (***A***) where genomic data might be less informative. It ensures that information is not lost from either data source. As noticed in the wheat datasets, this combination has resulted in a better genomic prediction accuracy, especially for individual sites. The combination provides a more balanced representation of genetic relationships, particularly when one matrix is less reliable for certain individuals or environments.

### 4.2 Positioning *C* and *P* within the machine learning landscape

The hybrid kernels ***C*** and ***P***, derived from the product of the genomic relationship matrix ***K*** and the pedigree matrix ***A***, share conceptual and computational parallels with modern machine learning frameworks such as Deep Learning (DL) and Graph Neural Networks (GNNs). Like DL models, these hybrid kernels can capture non-linear interactions and complex, higher-order patterns that arise from the integration of genomic and pedigree data. Furthermore, the structure of these kernel products resembles the message-passing mechanisms employed in GNNs, where information is propagated and aggregated across nodes connected in a graph, analogous to genetic relationships in a breeding population.

In this context, the matrix multiplication ***K*** × ***A*** can be interpreted as a biologically grounded form of message passing within a genetic network, where both realized relationships (captured by ***K***) and expected relationships (captured by ***A***) are integrated into a unified framework. This hybrid construction provides a meaningful and interpretable alternative to the opaquer representations typically found in DL and GNN approaches. By embedding domain-specific knowledge directly into the kernel structure, this method enhances the biological relevance of the model while maintaining or improving predictive performance.

Empirical results support the utility of this approach. In single-environment genomic prediction analyses, the proposed method (referred to as S5) consistently outperformed traditional baseline models (S1 through S4). For instance, using the Wheat 599 dataset, the S5 model yielded predictive correlations of 0.676, 0.614, 0.554, and 0.662 across four individual environments. These results compare favorably with those reported by Kihlman et al. (2024), who achieved an average predictive correlation of 0.456 using a GNN approach applied to the average phenotypic values across the same four environments.

### 4.3 The new method under G×E

This innovative method of combining matrices could also help with genotype-by-environment interaction (G×E) models, although, as observed, the improvement there might be less pronounced, particularly when information for the environment to be predicted is scarce. This could be due to GE interactions often requiring finer resolution in terms of environmental covariates, which neither ***A*** nor ***K*** alone can fully capture.

The reduction in the benefit of combining matrix ***G*** with matrix ***A*** when incorporating GE into the model can be attributed to several factors: (1) GE occurs when the effect of genotypes varies depending on environmental conditions, making it difficult for a single hybrid matrix to model all possible genotype responses. (2) GE introduces additional variance not accounted for by additive genetic models, potentially diminishing the effectiveness of ***A***, ***K***, or their combination. (3) Accurate G×E modeling may require detailed environment-specific information for each genotype, which hybrid kernels may not sufficiently represent. (4) The combination of ***A*** and ***K*** assumes additive, independent contributions. In contrast, GE often involves complex interactions that violate this assumption. (5) GE may involve non-linear patterns that are difficult to capture with linear kernels or additive frameworks. (6) Beyond additive effects, GE may include epistatic interactions that neither ***A*** nor ***K*** is designed to capture effectively.

Nevertheless, the proposed method is better when more phenotypic data from the environment to be predicted is available. One possible way to improve these predictions in the absence of phenotypic data from the environment to be predicted is to use the correlation matrix ***E*** between environments predicted with environmental and agronomic covariates, and then use it as the covariance matrix for the GE effects $\sigma_{g_{A\;}}^{2}Z_{1}AZ_{1}^{'}\circ Z_{2}EZ_{2\;}^{'}$ . The above could be a research alternative.

Although the measurements in the following cases are not directly comparable, it can be observed that for GE models with M5, the results are better than when using GE with Deep Learning on wheat data, as in Crossa et al. (2024).

### 4.4 Benefits of combining matrices *A* and *K*

Several studies in literature have demonstrated the advantages of jointly modeling pedigree and marker information to improve genomic prediction. For example, de los Campos et al. (2009), using both wheat and mouse datasets, found that models incorporating both pedigree and marker data outperformed those using only one source. Similarly, Crossa et al. (2014) showed that combining the pedigree matrix (***A***) and the genomic kernel (***K***) resulted in superior predictive ability compared to models using ***A*** or ***K*** alone. This improvement was consistent across different cross-validation strategies (CV1 and CV2) for grain yield prediction in wheat. Similarly to our strategy Velazco et al. (2019) proposed a weighted combination of pedigree and genomic relationship matrices, defined as: $K=wA+\left(1-w\right)G_{s}$ where K is their proposed matrix, A is the pedigree-based relationship matrix, $G_{s}$ is the rescaled genomic relationship matrix and w is a weighting factor. In line with our findings, they reported that this integrated matrix improved prediction performance compared to standard G-BLUP across various traits and prediction scenarios in sorghum breeding.

The proposed methodology of combining matrices ***A*** and ***K*** offers several significant advantages for the optimization of genomic prediction. The hybrid matrix effectively captures both expected and realized genetic relationships, providing a more holistic view of genetic variance within a breeding population. Moreover, combining ***A*** and ***K*** is particularly effective for complex traits where marker data alone cannot fully capture the underlying additive genetic variation. By integrating pedigree-derived relationships with genomic similarities, this approach helps to better understand how traits are inherited across generations while accounting for the actual genetic diversity present.

The hybrid approach allows for flexibility in model specifications, making it adaptable to various breeding objectives and environmental conditions. By adjusting the emphasis on either matrix based on the available data or specific research questions, breeders can tailor the approach to meet their unique needs. This adaptability extends the applicability of the methodology beyond wheat breeding to other crops and livestock species.

While incorporating G×E may present challenges, the hybrid matrix provides a framework for investigating how genotypes respond to varying environmental conditions. By using both pedigree and genomic data, breeders can gain insights into the stability of traits across environments and identify superior genotypes that perform consistently well under diverse conditions.

The proposed combination of matrices serves as a foundation for further methodological advances in genomic prediction. Future research could explore the integration of additional data types, such as phenotypic measurements or environmental covariates, to enhance the predictive power of the model. Additionally, examining the interaction between G×E and the combined matrix can lead to new insights and applications in breeding programs.

In summary, models S5 and M5 are recommended when both genomic and pedigree data are available and when predictive accuracy is a priority. Simpler models (S1, M1) may be used for exploratory analyses or when data are limited. The hybrid models offer a robust, biologically informed alternative to purely genomic or pedigree-based approaches and should be considered especially in complex breeding contexts with genotype-by-environment interactions (**Table 2**).

### 4.5 Why combine *K* and *A*?

While genomic relationship matrix (***K***) captures realized relationships via marker data (identity-by-state, IBS), the pedigree matrix (***A***) represents expected relationships via recorded ancestry (identity-by-descent, IBD). These two perspectives provide complementary information. Empirical studies (e.g., Crossa et al., 2010; Legarra et al., 2009) have shown that their joint use enhances prediction accuracy

The product ***K* × *A*** in our study is a matrix multiplication, not a Hadamard (elementwise) product. This operation results in a structured smoothing across genotypes and should be interpreted as a projection or convolution-like operation

The genomic matrix ***K*** captures realized relationships, while ***A*** captures expected relationships. Combining them allows the model to leverage both historical breeding information (pedigree) and observed genetic variation (genomic markers). The multiplication of ***K*** and ***A*** creates an interaction between these two types of information, generating new kernels (***C*** and ***P***) that introduce additional genetic variance components.

Model S5 extends Model S4 by including the ***C*** and ***P*** matrices in addition to the pedigree (***A***) and genomic (***K***) matrices. These additional covariance structures help capture genetic relationships that are neither fully explained by ***A*** nor ***K*** alone. Mathematically, ***C*** and ***P*** are transformations of ***KA***, where: ***C*** is the upper-triangle symmetric matrix capturing genomic variation weighted by pedigree. ***P*** is the lower-triangle symmetric matrix capturing pedigree relationships weighted by genomic similarity.

In breeding, genetic relationships are not strictly additive. Epistatic interactions, recombination, and selection history influence how traits are inherited. Matrices ***C*** and ***P*** capture additional genetic variance, allowing for better prediction accuracy. The fact that random effects modeled using ***C*** and ***P*** have low correlation with those modeled by ***A*** and ***K*** suggests that they provide independent genetic signals, which improve genomic prediction.

### 4.6 Biological advantages of model S5 in genomic prediction

As also noted by Howard et al. (2019), although it is biologically challenging to precisely interpret the variation captured by the product ***A* ∘*G*** or ***G*** ∘ ***A*** compared to ***A*** ∘ ***A*** or ***G*** ∘ ***G***, mathematically it reflects epistatic interactions—representing the interplay between marker and pedigree information rather than relying solely on marker-based relatedness. Anyway, Model S5 enhances genomic prediction by integrating both pedigree-based (***A***) and genomic-based (***K***) relationship matrices while introducing two additional covariance structures (***C*** and ***P***). These modifications offer several key biological advantages: (1) captures realized (***K***) and expected genetic relationships (***A***) and by combining them, Model S5 accounts for both inheritance history and observed genetic variation, leading to more biologically meaningful predictions; (2) models complex genetic architecture beyond additive effects as traditional models assume additive genetic effects, but biological traits are influenced by epistasis, recombination, and selection history. The ***C*** matrix (upper-triangle transformation of ***KA***) and ***P*** matrix (lower-triangle transformation of ***KA***) capture interactions between pedigree and genomic information, introducing new genetic variance components; (3) Improves detection of independent genetic signals as the low correlation between random effects modeled by ***A***/***K*** and ***C***/***P*** suggests that these new matrices capture additional independent genetic information. This implies that genetic signals missed by ***A*** and ***K*** alone are now included, enhancing predictive accuracy; (4) decisions by incorporating ***C*** and ***P***, Model S5 provides a more refined genetic variance structure, which helps breeders to identify hidden genetic potential in populations (5) accounts for selection history and recombination effects by weighting genomic relationships with pedigree (***C***) and vice versa (***P***), Model S5 captures how past breeding decisions shaped the current genetic structure of a population.

In summary, model S5 improves genomic prediction by capturing additional genetic variance components (beyond ***A*** and ***K***) allowing for a more biologically realistic representation of genetic inheritance. By integrating historical and realized genetic relationships while incorporating non-additive interactions, it enhances prediction accuracy and provides better insights for breeding strategies.

### 4.7 Real-world breeding considerations

The effectiveness of a breeding program is often evaluated by the rate of genetic gain per cycle, represented by the equation: $\Delta G=\frac{ir\sigma_{a}}{L}$ where i is the selection intensity, r is the accuracy of the selection, $\sigma_{a}$ is the square root of the additive genetic variance, and L is the cycle time express in year number (Eberhart, 1970; Cobb et al., 2019). In traditional breeding programs, the selection accuracy ( r ) is typically defined as the correlation between true breeding values (TBVs) and estimated breeding values (EBVs), which can be approximated by the square root of the narrow-sense heritability (Cobb et al., 2019). In contrast, in genomic selection (GS)-based programs, r is defined as the correlation between TBVs and genomic estimated breeding values (GEBVs) (Lorenz, 2013; Xu et al., 2017). Since TBVs are unobservable in practice, model predictability, measured as the correlation between GEBVs and observed phenotypes such as BLUEs, is often used as a proxy for r .

Improving model predictability is therefore essential in GS-based breeding, as it directly impacts the rate of genetic gain and, consequently, the overall success of the program. In CIMMYT’s wheat breeding pipeline, genomic prediction models are typically trained on five years of Elite Yield Trial (EYT) data and used to predict performance in a target population consisting of pre-yield, untested lines. Breeders then make selection decisions based on the resulting GEBVs.

Real-world validation across six simulated environments revealed low to moderate predictability for grain yield (Vitale et al., 2025), indicating substantial opportunity for optimization. One promising strategy explored in this study involves the combined use of the genomic relationship matrix (***K***) and the pedigree-based relationship matrix (***A***). This integrative approach demonstrated improved model predictability, offering a practical pathway to enhance the rate of genetic gain in wheat breeding programs. Despite the positive results observed in this study, further analyses using additional and diverse datasets are required to examine the generalizability and robustness of these findings

## 5 Conclusion

The integration of pedigree and genomic matrices has shown promising results in optimizing genomic prediction accuracy for wheat. By strategically combining the genomic matrix (***K***) with the pedigree matrix (***A***), and vice versa, we observed significant improvements in prediction accuracy across various datasets. This innovative approach not only leverages the strengths of both matrices but also addresses the complexities associated with genotype-by-environment interactions (G×E). While the increase in accuracy for individual sites was pronounced, the effects on G×E interactions were less pronounced, indicating the need for further exploration of how these interactions can be better modeled within combined frameworks. Overall, this study underscores the potential of matrix fusion strategies in advancing genomic selection methodologies and enhancing the predictive capabilities for breeding programs, particularly in the context of developing resilient wheat varieties suitable for diverse environmental conditions.

## Data Availability

The original contributions presented in the study are publicly available. This data can be found here: https://hdl.handle.net/11529/10549160.
