# Supplementary material for: Enhancing wheat genomic prediction by a hybrid kernel approach
Source: Front Plant Sci. 2025 Aug 1;16:1605202. doi: 10.3389/fpls.2025.1605202 (PMC12363290; doi:10.3389/fpls.2025.1605202)
Supplement: Supplementary file 1 [file SupplementaryFile1.docx]

Supplementary Material: Enhancing Wheat Genomic Prediction by a Hybrid Kernel Approach

Jaime Cuevas^1^, Jose Crossa^2,3^, Abelardo Montesinos-López^4^, Johannes W. R. Martini^5^, Guillermo Gerard^2^, Jaime Ortegón^1^, Susanne Dreisigacker^2^, Velu, Govindan^2^, Zerihun Tadesse^2^, Paulino Pérez-Rodríguez^3^, Carolina Saint Pierre^2^, Leonardo Crespo Herrera^2^, Osval A. Montesinos-López^6^*, Paolo Vitale^2^*

^1^División de Ciencias, Ingeniería y Tecnologías (DCIT), Universidad Autónoma del Estado de Quintana Roo, Chetumal, Quintana Roo, México.

^2^International Maize and Wheat Improvement Center (CIMMYT), Km 45, Carretera Mexico-Veracruz, CP 52640, Edo. de México, México.

^3^Colegio de Postgraduados, Montecillos, Edo. de México CP 56230, México.

^4^Centro Universitario de Ciencias Exactas e Ingenierías (CUCEI), Universidad de Guadalajara, 44430, Guadalajara, Jalisco, México.

^5^Aardevo B.V., Nagele, The Netherlands

^6^Facultad de Telemática, Universidad de Colima, Colima, Colima, 28040, México.

*** Correspondence:**Corresponding Author
[osval78t@gmail.com](mailto:osval78t@gmail.com)**,** [p.vitale@cgiar.org](mailto:p.vitale@cgiar.org)

# Supplementary Data

Table S1. General data information

| **Dataset** | **Enviroment** | **# Observations** | **Mean** | **SD** | **Order**  **Pedigree Matrix** | **Order**  **Markers Matrix** |
| --- | --- | --- | --- | --- | --- | --- |
| EYT_2016_2017 | B2IR_16_17 | 1092 | 4.83 | 0.43 | 1092 x1092 | 1092x3714 |
|  | B5IR_16_17 | 1092 | 6.5 | 0.56 |  |  |
|  | BEHT_16_17 | 1092 | 6.27 | 0.73 |  |  |
|  | BLHT_16_17 | 1092 | 2.62 | 0.5 |  |  |
|  | DRT_16_17 | 1092 | 3.95 | 0.45 |  |  |
|  | F5IR_16_17 | 1092 | 6.29 | 0.69 |  |  |
| EYT_2022_2023 | B2IR_22_23 | 1004 | 5.24 | 0.43 | 1004x1004 | 1004x3672 |
|  | B5IR_22_23 | 1004 | 8.1 | 0.51 |  |  |
|  | BDRT_22_23 | 1004 | 4.01 | 0.28 |  |  |
|  | BEHT_22_23 | 1004 | 7.89 | 0.51 |  |  |
|  | BLHT_22_23 | 1004 | 7.14 | 0.55 |  |  |
|  | F5IR_22_23 | 1004 | 7.93 | 0.48 |  |  |
| EYT_2023_2024 | B2IR_23_24 | 898 | 5.32 | 0.37 | 898x898 | 898x3267 |
|  | B5IR_23_24 | 898 | 7.85 | 0.51 |  |  |
|  | BEHT_23_24 | 898 | 7.76 | 0.42 |  |  |
|  | BLHT_23_24 | 898 | 6.11 | 0.45 |  |  |
|  | F5IR_23_24 | 898 | 8.52 | 0.46 |  |  |
| Wheat 599 | E1 | 599 | 0 | 1 | 599x599 | 599x1271 |
|  | E2 | 599 | 0 | 1 |  |  |
|  | E3 | 599 | 0 | 1 |  |  |
|  | E4 | 599 | 0 | 1 |  |  |

Table S2. Correlations between the predicted vectors of model S5 in the data sets EYT-16_17 and EYT_22_23

|  | | | | | | | |
| --- | --- | --- | --- | --- | --- | --- | --- |
| EYT_16_17 | | | | EYT_22_23 | | | |
| Trait |  | $\boldsymbol{g}_{C}$ | $\boldsymbol{g}_{P}$ | Trait |  | $\boldsymbol{g}_{C}$ | $\boldsymbol{g}_{P}$ |
| DRT_16_17 | $\boldsymbol{g}_{A}$ | 0.23 | 0.23 | B2IR_22_23 | $\boldsymbol{g}_{A}$ | 0.23 | 0.21 |
|  | $\boldsymbol{g}_{K}$ | 0.20 | 0.23 |  | $\boldsymbol{g}_{K}$ | 0.18 | 0.17 |
|  | $\boldsymbol{g}_{C}$ | 1.00 | 0.02 |  | $\boldsymbol{g}_{C}$ | 1.00 | 0.02 |
|  | $\boldsymbol{g}_{P}$ | 0.02 | 1.00 |  | $\boldsymbol{g}_{P}$ | 0.02 | 1.00 |
| BEHT_16_17 | $\boldsymbol{g}_{A}$ | 0.35 | 0.08 | B5IR_22_23 | $\boldsymbol{g}_{A}$ | 0.12 | 0.11 |
|  | $\boldsymbol{g}_{K}$ | 0.33 | 0.25 |  | $\boldsymbol{g}_{K}$ | 0.28 | 0.14 |
|  | $\boldsymbol{g}_{C}$ | 1.00 | -0.02 |  | $\boldsymbol{g}_{C}$ | 1.00 | 0.06 |
|  | $\boldsymbol{g}_{P}$ | -0.02 | 1.00 |  | $\boldsymbol{g}_{P}$ | 0.06 | 1.00 |
| F5IR_16_17 | $\boldsymbol{g}_{A}$ | 0.26 | 0.18 | BDRT_22_23 | $\boldsymbol{g}_{A}$ | 0.29 | 0.19 |
|  | $\boldsymbol{g}_{K}$ | 0.26 | 0.25 |  | $\boldsymbol{g}_{K}$ | 0.21 | 0.20 |
|  | $\boldsymbol{g}_{C}$ | 1.00 | 0.04 |  | $\boldsymbol{g}_{C}$ | 1.00 | 0.04 |
|  | $\boldsymbol{g}_{P}$ | 0.04 | 1.00 |  | $\boldsymbol{g}_{P}$ | 0.04 | 1.00 |
| B2IR_16_17 | $\boldsymbol{g}_{A}$ | 0.26 | 0.39 | BEHT_22_23 | $\boldsymbol{g}_{A}$ | 0.16 | 0.12 |
|  | $\boldsymbol{g}_{K}$ | 0.33 | 0.30 |  | $\boldsymbol{g}_{K}$ | 0.15 | 0.22 |
|  | $\boldsymbol{g}_{C}$ | 1.00 | 0.02 |  | $\boldsymbol{g}_{C}$ | 1.00 | 0.00 |
|  | $\boldsymbol{g}_{P}$ | 0.02 | 1.00 |  | $\boldsymbol{g}_{P}$ | 0.00 | 1.00 |
| BLHT_16_17 | $\boldsymbol{g}_{A}$ | 0.30 | 0.23 | F5IR_23_24 | $\boldsymbol{g}_{A}$ | 0.07 | -0.01 |
|  | $\boldsymbol{g}_{K}$ | 0.27 | 0.23 |  | $\boldsymbol{g}_{K}$ | 0.16 | 0.16 |
|  | $\boldsymbol{g}_{C}$ | 1.00 | -0.03 |  | $\boldsymbol{g}_{C}$ | 1.00 | 0.05 |
|  | $\boldsymbol{g}_{P}$ | -0.03 | 1.00 |  | $\boldsymbol{g}_{P}$ | 0.05 | 1.00 |
| B5IR_16_17 | $\boldsymbol{g}_{A}$ | 0.25 | 0.28 | BLHT_23_24 | $\boldsymbol{g}_{A}$ | 0.25 | 0.20 |
|  | $\boldsymbol{g}_{K}$ | 0.31 | 0.30 |  | $\boldsymbol{g}_{K}$ | 0.21 | 0.26 |
|  | $\boldsymbol{g}_{C}$ | 1.00 | 0.05 |  | $\boldsymbol{g}_{C}$ | 1.00 | -0.05 |
|  | $\boldsymbol{g}_{P}$ | 0.05 | 1.00 |  | $\boldsymbol{g}_{P}$ | -0.05 | 1.00 |

Table S3. Correlations between predicted vectors GC and GP of model S5 in data sets EYT-23_24 and EYT_599

| EYT_23_24 | | | | EYT-599 | | | |
| --- | --- | --- | --- | --- | --- | --- | --- |
| Trait |  | $\boldsymbol{g}_{C}$ | $\boldsymbol{g}_{P}$ | Trait |  | $\boldsymbol{g}_{C}$ | $\boldsymbol{g}_{P}$ |
| B2IR_23_24 | $\boldsymbol{g}_{A}$ | 0.10 | 0.15 | E1(599) | $\boldsymbol{g}_{A}$ | 0.24 | 0.11 |
|  | $\boldsymbol{g}_{K}$ | 0.19 | 0.16 |  | $\boldsymbol{g}_{K}$ | 0.23 | 0.28 |
|  | $\boldsymbol{g}_{C}$ | 1.00 | -0.01 |  | $\boldsymbol{g}_{C}$ | 1.00 | 0.18 |
|  | $\boldsymbol{g}_{P}$ | -0.01 | 1.00 |  | $\boldsymbol{g}_{P}$ | 0.18 | 1.00 |
| B5IR_23_24 | $\boldsymbol{g}_{A}$ | 0.08 | 0.19 | E2(599) | $\boldsymbol{g}_{A}$ | 0.30 | 0.36 |
|  | $\boldsymbol{g}_{K}$ | 0.17 | 0.13 |  | $\boldsymbol{g}_{K}$ | 0.25 | 0.31 |
|  | $\boldsymbol{g}_{C}$ | 1.00 | -0.02 |  | $\boldsymbol{g}_{C}$ | 1.00 | 0.09 |
|  | $\boldsymbol{g}_{P}$ | -0.02 | 1.00 |  | $\boldsymbol{g}_{P}$ | 0.09 | 1.00 |
| BEHT_23_24 | $\boldsymbol{g}_{A}$ | 0.25 | 0.16 | E3(599) | $\boldsymbol{g}_{A}$ | 0.27 | 0.16 |
|  | $\boldsymbol{g}_{K}$ | 0.14 | 0.25 |  | $\boldsymbol{g}_{K}$ | 0.33 | 0.31 |
|  | $\boldsymbol{g}_{C}$ | 1.00 | 0.02 |  | $\boldsymbol{g}_{C}$ | 1.00 | 0.20 |
|  | $\boldsymbol{g}_{P}$ | 0.02 | 1.00 |  | $\boldsymbol{g}_{P}$ | 0.20 | 1.00 |
| BLHT_23_24 | $\boldsymbol{g}_{A}$ | 0.05 | 0.24 | E4(599) | $\boldsymbol{g}_{A}$ | 0.02 | 0.29 |
|  | $\boldsymbol{g}_{K}$ | 0.12 | 0.18 |  | $\boldsymbol{g}_{K}$ | 0.08 | 0.32 |
|  | $\boldsymbol{g}_{C}$ | 1.00 | 0.01 |  | $\boldsymbol{g}_{C}$ | 1.00 | -0.02 |
|  | $\boldsymbol{g}_{P}$ | 0.01 | 1.00 |  | $\boldsymbol{g}_{P}$ | -0.02 | 1.00 |
| F5IR_23_24 | $\boldsymbol{g}_{A}$ | 0.30 | 0.03 |  |  |  |  |
|  | $\boldsymbol{g}_{K}$ | 0.20 | 0.18 |  |  |  |  |
|  | $\boldsymbol{g}_{C}$ | 1.00 | 0.04 |  |  |  |  |
|  | $\boldsymbol{g}_{P}$ | 0.04 | 1.00 |  |  |  |  |

Table S4. Average PMSE and SD for EYT_16_17 and EYT _22_23 for five single-environment models using five- and two-fold cross-validation strategies. “Diff” corresponds to the difference between the standard GBLUP (M2) and the proposed model (M5).

|  | **Five-fold Cross-validation** | | | | | | | **Two-fold cross-validation** | | | | | |
| --- | --- | --- | --- | --- | --- | --- | --- | --- | --- | --- | --- | --- | --- |
| **Data set** |  | **S1** | **S2** | **S3** | **S4** | **S5** | **Diff** | **S1** | **S2** | **S3** | **S4** | **S5** | **Diff** |
| **B2IR_16_17** | AVG | 0.118 | 0.125 | 0.116 | 0.108 | **0.101** | -19% | 0.125 | 0.133 | 0.126 | 0.117 | **0.112** | -16% |
|  | SD | 0.012 | 0.013 | 0.012 | 0.010 | 0.008 |  | 0.005 | 0.005 | 0.005 | 0.004 | 0.004 |  |
| **B5IR_16_17** | AVG | 0.205 | 0.227 | 0.210 | 0.194 | **0.174** | -23% | 0.219 | 0.235 | 0.222 | 0.207 | **0.193** | -18% |
|  | SD | 0.015 | 0.023 | 0.022 | 0.016 | 0.012 |  | 0.012 | 0.011 | 0.011 | 0.010 | 0.012 |  |
| **BEHT_16_17** | AVG | 0.362 | 0.372 | 0.351 | 0.333 | **0.322** | -13% | 0.377 | 0.386 | 0.369 | 0.353 | **0.345** | -11% |
|  | SD | 0.047 | 0.038 | 0.040 | 0.042 | 0.039 |  | 0.018 | 0.016 | 0.015 | 0.017 | 0.017 |  |
| **BLHT_16_17** | AVG | 0.182 | 0.189 | 0.174 | 0.163 | **0.154** | -19% | 0.196 | 0.200 | 0.189 | 0.180 | **0.170** | -15% |
|  | SD | 0.022 | 0.018 | 0.017 | 0.018 | 0.019 |  | 0.007 | 0.009 | 0.009 | 0.008 | 0.007 |  |
| **DRTT_16_17** | AVG | 0.144 | 0.160 | 0.151 | 0.138 | **0.120** | -25% | 0.159 | 0.169 | 0.162 | 0.154 | **0.134** | -21% |
|  | SD | 0.022 | 0.020 | 0.021 | 0.021 | 0.018 |  | 0.013 | 0.013 | 0.013 | 0.013 | 0.012 |  |
| **F5IR_16_17** | AVG | 0.339 | 0.308 | 0.292 | 0.289 | **0.279** | -9% | 0.350 | 0.327 | 0.317 | 0.314 | **0.304** | -7% |
|  | SD | 0.033 | 0.033 | 0.033 | 0.031 | 0.029 |  | 0.016 | 0.014 | 0.015 | 0.015 | 0.015 |  |
| **B2IR_22_23** | AVG | 0.203 | 0.235 | 0.233 | 0.229 | **0.212** | -10% | 0.169 | 0.167 | 0.166 | 0.164 | **0.142** | -15% |
|  | SD | 0.019 | 0.022 | 0.022 | 0.023 | 0.019 |  | 0.012 | 0.011 | 0.011 | 0.011 | 0.011 |  |
| **B5IR_22_23** | AVG | 0.203 | 0.235 | 0.233 | 0.229 | **0.212** | -10% | 0.247 | 0.240 | 0.237 | 0.235 | **0.213** | -11% |
|  | SD | 0.019 | 0.022 | 0.022 | 0.023 | 0.019 |  | 0.016 | 0.014 | 0.014 | 0.014 | 0.014 |  |
| **BDRT_22_23** | AVG | 0.065 | 0.074 | 0.073 | 0.072 | **0.068** | -8% | 0.076 | 0.075 | 0.074 | 0.073 | **0.068** | -9% |
|  | SD | 0.008 | 0.009 | 0.009 | 0.008 | 0.008 |  | 0.003 | 0.004 | 0.004 | 0.004 | 0.004 |  |
| **BEHT_22_23** | AVG | 0.212 | 0.221 | 0.219 | 0.219 | **0.214** | -3% | 0.240 | 0.226 | 0.225 | 0.224 | **0.221** | -2% |
|  | SD | 0.022 | 0.023 | 0.023 | 0.023 | 0.022 |  | 0.009 | 0.009 | 0.009 | 0.009 | 0.009 |  |
| **BLHT_22_23** | AVG | 0.292 | 0.285 | 0.284 | 0.282 | **0.282** | -1% | 0.240 | 0.226 | 0.225 | 0.224 | **0.288** | 27% |
|  | SD | 0.027 | 0.026 | 0.026 | 0.026 | 0.026 |  | 0.009 | 0.009 | 0.009 | 0.009 | 0.015 |  |
| **F5IR_22_23** | AVG | 0.209 | 0.220 | 0.219 | 0.207 | **0.214** | -3% | 0.240 | 0.226 | 0.225 | 0.224 | **0.186** | -18% |
|  | SD | 0.017 | 0.020 | 0.020 | 0.017 | 0.022 |  | 0.009 | 0.009 | 0.009 | 0.009 | 0.009 |  |

Table S5. Average PMSE and SD for EYT_23_24 and wheat_599 for five single-environment models using five- and two-fold cross-validation strategies. “Diff” corresponds to the difference between the standard GBLUP (M2) and the proposed model (M5).

|  | **Five-fold Cross-validation** | | | | | | | **Two-fold cross-validation** | | | | | |
| --- | --- | --- | --- | --- | --- | --- | --- | --- | --- | --- | --- | --- | --- |
| **Data set** |  | **S1** | **S2** | **S3** | **S4** | **S5** | **Diff** | **S1** | **S2** | **S3** | **S4** | **S5** | **Diff** |
| **B2IR_23_24** | AVG | 0.134 | 0.118 | 0.118 | 0 | **0.115** | -3% | 0.134 | 0.12 | 0.12 | 0.121 | **0.119** | -1% |
|  | SD | 0.009 | 0.009 | 0.009 | 0 | 0.01 |  | 0.007 | 0.006 | 0.006 | 0.006 | 0.006 |  |
| **B5IR_23_24** | AVG | 0.259 | 0.257 | 0.255 | 0.256 | **0.249** | -3% | 0.261 | 0.259 | 0.257 | 0.257 | **0.253** | -2% |
|  | SD | 0.026 | 0.026 | 0.027 | 0.027 | 0.025 |  | 0.01 | 0.01 | 0.01 | 0.01 | 0.01 |  |
| **BLHT_23_24** | AVG | 0.203 | 0.185 | 0.182 | 0.183 | **0.175** | -5% | 0.261 | 0.259 | 0.257 | 0.257 | **0.184** | -29% |
|  | SD | 0.02 | 0.018 | 0.019 | 0.019 | 0.017 |  | 0.01 | 0.01 | 0.01 | 0.01 | 0.014 |  |
| **F5IR_23_24** | AVG | 0.206 | 0.201 | 0.2 | 0.2 | **0.198** | -1% | 0.208 | 0.203 | 0.202 | 0.203 | **0.201** | -1% |
|  | SD | 0.017 | 0.018 | 0.018 | 0.017 | 0.016 |  | 0.01 | 0.009 | 0.009 | 0.01 | 0.01 |  |
| **E1 (599)** | AVG | 0.827 | 0.75 | 0.68 | 0.672 | **0.545** | -27% | 0.865 | 0.794 | 0.733 | 0.72 | **0.577** | -27% |
|  | SD | 0.101 | 0.087 | 0.101 | 0.097 | 0.081 |  | 0.059 | 0.052 | 0.053 | 0.054 | 0.039 |  |
| **E2 (599)** | AVG | 0.843 | 0.755 | 0.749 | 0.75 | **0.625** | -17% | 0.872 | 0.81 | 0.807 | 0.809 | **0.678** | -16% |
|  | SD | 0.084 | 0.082 | 0.08 | 0.082 | 0.08 |  | 0.055 | 0.055 | 0.056 | 0.05 | 0.052 |  |
| **E3 (599)** | AVG | 0.849 | 0.855 | 0.826 | 0.805 | **0.694** | -19% | 0.88 | 0.886 | 0.866 | 0.845 | **0.739** | -17% |
|  | SD | 0.137 | 0.153 | 0.141 | 0.135 | 0.11 |  | 0.053 | 0.052 | 0.053 | 0.052 | 0.048 |  |
| **E4 (599)** | AVG | 0.809 | 0.801 | 0.744 | 0.732 | **0.567** | -29% | 0.851 | 0.84 | 0.798 | 0.796 | **0.607** | -28% |
|  | SD | 0.096 | 0.075 | 0.079 | 0.089 | 0.069 |  | 0.05 | 0.05 | 0.046 | 0.051 | 0.048 |  |

Table S6. Average PMSE and SD for EYT_16_17 and EYT _22_23 for five multi-environment models using five- and two-fold cross-validation strategies. “Diff” corresponds to the difference between the standard GBLUP (M2) and the proposed model (M5).

|  | **Five-fold Cross-validation** | | | | | | | **Two-fold cross-validation** | | | | | |
| --- | --- | --- | --- | --- | --- | --- | --- | --- | --- | --- | --- | --- | --- |
| **Data set** |  | **M1** | **M2** | **M3** | **M4** | **M5** | **Diff** | **M1** | **M2** | **M3** | **M4** | **M5** | **Diff** |
| **B2IR_16_17** | AVG | 0.132 | 0.130 | 0.123 | 0.124 | **0.111** | -14% | 0.158 | 0.155 | **0.151** | 0.152 | 0.148 | -4% |
|  | SD | 0.006 | 0.004 | 0.004 | 0.004 | 0.003 |  | 0.013 | 0.013 | 0.015 | 0.015 | 0.014 |  |
| **B5IR_16_17** | AVG | 0.251 | 0.251 | 0.248 | 0.249 | **0.2127** | -15% | 0.298 | 0.290 | 0.297 | **0.295** | 0.279 | -4% |
|  | SD | 0.010 | 0.011 | 0.011 | 0.010 | 0.011 |  | 0.017 | 0.013 | 0.016 | 0.014 | 0.009 |  |
| **BEHT_16_17** | AVG | 0.406 | 0.417 | 0.408 | 0.408 | **0.369** | -12% | 0.478 | 0.471 | 0.477 | 0.481 | **0.471** | 0% |
|  | SD | 0.011 | 0.014 | 0.017 | 0.017 | 0.017 |  | 0.028 | 0.023 | 0.024 | 0.024 | 0.037 |  |
| **BLHT_16_17** | AVG | 0.209 | 0.212 | 0.203 | 0.203 | **0.1869** | -12% | 0.235 | 0.232 | 0.231 | 0.231 | **0.224** | -3% |
|  | SD | 0.007 | 0.010 | 0.010 | 0.011 | 0.005 |  | 0.014 | 0.012 | 0.013 | 0.014 | 0.011 |  |
| **BDRT_16_17** | AVG | 0.164 | 0.165 | 0.154 | 0.154 | **0.127** | -23% | 0.183 | 0.178 | 0.169 | 0.170 | **0.159** | -11% |
|  | SD | 0.007 | 0.008 | 0.007 | 0.008 | 0.007 |  | 0.005 | 0.007 | 0.006 | 0.006 | 0.009 |  |
| **F5IR_16_17** | AVG | 0.347 | 0.329 | 0.322 | 0.320 | **0.31** | -6% | 0.398 | 0.374 | 0.380 | 0.377 | **0.377** | 1% |
|  | SD | 0.013 | 0.016 | 0.015 | 0.015 | 0.016 |  | 0.015 | 0.020 | 0.023 | 0.020 | 0.018 |  |
| **B2IR_22_23** | AVG | 0.1453 | 0.147 | 0.1375 | 0.137 | **0.125** | -15% | 0.153 | 0.154 | 0.146 | 0.148 | **0.144** | -6% |
|  | SD | 0.005 | 0.005 | 0.004 | 0.004 | 0.005 |  | 0.007 | 0.008 | 0.009 | 0.007 | 0.009 |  |
| **B5IR_22_23** | AVG | 0.2288 | 0.2268 | 0.217 | 0.217 | **0.1896** | -16% | 0.239 | 0.234 | 0.228 | 0.227 | **0.21** | -10% |
|  | SD | 0.009 | 0.005 | 0.005 | 0.005 | 0.006 |  | 0.011 | 0.009 | 0.009 | 0.009 | 0.010 |  |
| **BDRT_22_23** | AVG | 0.074 | 0.0727 | 0.0709 | 0.0708 | **0.066** | -9% | 0.080 | 0.079 | 0.077 | 0.077 | **0.076** | -4% |
|  | SD | 0.003 | 0.003 | 0.003 | 0.003 | 0.003 |  | 0.004 | 0.004 | 0.003 | 0.002 | 0.004 |  |
| **BEHT_22_23** | AVG | 0.219 | 0.2115 | 0.201 | 0.2018 | **0.199** | -6% | 0.233 | 0.228 | 0.223 | **0.222** | 0.224 | -2% |
|  | SD | 0.007 | 0.005 | 0.005 | 0.005 | 0.007 |  | 0.009 | 0.010 | 0.012 | 0.013 | 0.009 |  |
| **BLHT_22_23** | AVG | 0.301 | 0.2928 | 0.286 | **0.285** | 0.2915 | 0% | 0.317 | 0.307 | 0.302 | **0.302** | 0.311 | 1% |
|  | SD | 0.007 | 0.008 | 0.007 | 0.007 | 0.008 |  | 0.015 | 0.011 | 0.012 | 0.012 | 0.012 |  |
| **F5IR_22_23** | AVG | 0.2207 | 0.226 | 0.2219 | 0.221 | **0.187** | -17% | 0.238 | 0.241 | 0.241 | 0.241 | **0.222** | -8% |
|  | SD | 0.005 | 0.006 | 0.007 | 0.006 | 0.009 |  | 0.009 | 0.007 | 0.008 | 0.009 | 0.010 |  |

Table S7. Average PMSE and SD for EYT_23_24 and wheat_599 for five multi-environment models using five- and two-fold cross-validation strategies. “Diff” corresponds to the difference between the standard GBLUP (M2) and the proposed model (M5).

|  | **Five-fold Cross-validation** | | | | | | | **Two-fold cross-validation** | | | | | |
| --- | --- | --- | --- | --- | --- | --- | --- | --- | --- | --- | --- | --- | --- |
| **Data set** |  | **M1** | **M2** | **M3** | **M4** | **M5** | **Diff** | **M1** | **M2** | **M3** | **M4** | **M5** | **Diff** |
| **B2IR_23_24** | AVG | 0.1317 | 0.12 | 0.118 | **0.117** | 0.118 | -2% | 0.137 | 0.125 | 0.125 | **0.125** | 0.131 | 5% |
|  | SD | 0.004 | 0.007 | 0.006 | 0.006 | 0.005 |  | 0.004 | 0.007 | 0.007 | 0.006 | 0.007 |  |
| **B5IR_23_24** | AVG | 0.255 | 0.252 | 0.241 | 0.241 | **0.234** | -7% | 0.263 | 0.263 | 0.256 | 0.257 | **0.252** | -4% |
|  | SD | 0.011 | 0.010 | 0.009 | 0.008 | 0.012 |  | 0.012 | 0.012 | 0.014 | 0.010 | 0.012 |  |
| **BEHT_23_24** | AVG | 0.1675 | 0.163 | 0.157 | 0.157 | **0.153** | -6% | 0.17 | 0.167 | 0.163 | 0.163 | **0.161** | -4% |
|  | SD | 0.004 | 0.007 | 0.007 | 0.007 | 0.006 |  | 0.003 | 0.004 | 0.007 | 0.008 | 0.007 |  |
| **BLHT_23_24** | AVG | 0.2117 | 0.196 | 0.192 | 0.193 | **0.19** | -3% | 0.215 | 0.21 | **0.209** | 0.21 | 0.211 | 0% |
|  | SD | 0.008 | 0.008 | 0.007 | 0.007 | 0.009 |  | 0.015 | 0.015 | 0.013 | 0.016 | 0.011 |  |
| **F5IR_23_24** | AVG | 0.2 | 0.2 | 0.191 | 0.192 | **0.188** | -6% | 0.221 | 0.205 | 0.201 | 0.2 | **0.199** | -3% |
|  | SD | 0.007 | 0.009 | 0.007 | 0.009 | 0.008 |  | 0.007 | 0.006 | 0.008 | 0.007 | 0.007 |  |
| **E1 (599)** | AVG | 0.909 | 0.854 | 0.827 | 0.83 | **0.682** | -20% | 0.959 | 0.965 | 0.947 | 0.946 | **0.852** | -12% |
|  | SD | 0.040 | 0.030 | 0.032 | 0.033 | 0.034 |  | 0.055 | 0.039 | 0.041 | 0.045 | 0.038 |  |
| **E2 (599)** | AVG | 0.623 | 0.672 | 0.594 | 0.592 | **0.523** | -22% | 0.66 | 0.73 | 0.648 | 0.652 | **0.595** | -18% |
|  | SD | 0.019 | 0.026 | 0.023 | 0.019 | 0.015 |  | 0.078 | 0.071 | 0.077 | 0.077 | 0.078 |  |
| **E3 (599)** | AVG | 0.656 | 0.711 | 0.618 | 0.619 | **0.57** | -20% | 0.741 | 0.78 | 0.711 | 0.712 | **0.692** | -11% |
|  | SD | 0.042 | 0.033 | 0.029 | 0.030 | 0.037 |  | 0.093 | 0.060 | 0.070 | 0.071 | 0.067 |  |
| **E4 (599)** | AVG | 0.809 | 0.814 | 0.776 | 0.777 | 0.681 | -16% | 0.868 | 0.889 | 0.87 | 0.869 | **0.814** | -8% |
|  | SD | 0.027 | 0.029 | 0.028 | 0.031 | 0.029 |  | 0.050 | 0.072 | 0.067 | 0.068 | 0.064 |  |

**Box 1**

| #computing KA  KA=K%*%A  #computing KA diagonal  DKA=diag(KA)  #computing the upper triangle (UT)  UT=***KA***  UT[!upper.tri(***KA***)] <- 0  #computing the lower triangle (LT)  LT=***KA***  LT[!lower.tri(***KA***)] <- 0  #computing C  ***C***=UT+t(UT)  diag(***C***)=diag(***KA***)  **C**=***C***/mean(diag(***C***))  #computing P  ***P***=LT+t(LT)  diag(***P***)=diag(***KA***)  ***P***=***P***/mean(diag(***P***)) |
| --- |
